# Acute Kidney Injury Post-cardiac Surgery in Infants and Children: A Single-Center Experience in a Developing Country

**DOI:** 10.3389/fped.2021.637463

**Published:** 2021-07-26

**Authors:** Bilal Aoun, Ghadi Abu Daher, Karim N. Daou, Sami Sanjad, Hani Tamim, Issam El Rassi, Mariam Arabi, Rana Sharara, Fadi Bitar, Jana Assy, Ziad Bulbul, Jad A. Degheili, Marianne Majdalani

**Affiliations:** ^1^Division of Pediatric Nephrology, Department of Pediatrics and Adolescent Medicine, American University of Beirut Medical Center, Beirut, Lebanon; ^2^Division of Pediatric Intensive Care Unit, Department of Pediatrics and Adolescent Medicine, American University of Beirut Medical Center, Beirut, Lebanon; ^3^Biostatistics Unit, Faculty of Medicine, Clinical Research Institute, American University of Beirut, Beirut, Lebanon; ^4^Division of Cardiothoracic Surgery, Department of Surgery, American University of Beirut Medical Center, Beirut, Lebanon; ^5^Division of Pediatric Cardiology, Department of Pediatrics and Adolescent Medicine, American University of Beirut Medical Center, Beirut, Lebanon; ^6^Division of Urology, Department of Surgery, American University of Beirut Medical Center, Beirut, Lebanon

**Keywords:** acute kidney injury, pediatrics, cardiac surgery, pediatric intensive care unit, developing country

## Abstract

**Introduction:** The incidence of acute kidney injury (AKI) in pediatric patients following cardiac surgery varies between 15 and 64%, with a mortality rate of 10–89% among those requiring dialysis. This variation in the incidence and mortality of AKI across studies is probably due to the inconsistent definitions used for AKI. The purpose of this study is to present our experience with AKI post-cardiac surgery with emphasis on predisposing or aggravating factors.

**Patients and Methods:** We evaluated the incidence of AKI using the KDIGO criteria in 150 infants and children undergoing cardiac surgeries between 2015 and 2017. Post-operatively, all patients were admitted to the pediatric intensive care unit (PICU) at a tertiary care center in a developing country. This is a retrospective chart review in which data collected included age, gender, type of heart disease, prior cardiac surgeries, RACHS-1 category, and pre- and post-operative creatinine levels. Neonates were not included in this study.

**Results:** Six percent of the studied patients were below 1 year of age, 84% 1–10 years, and 10% 10–18 years. Fourteen patients (9.3%) developed AKI. Patients with cyanotic heart disease were more prone to develop AKI (78%) compared to those with non-cyanotic heart disease (44%). Children with AKI had a higher length of stay in PICU, 2.56 ± 1.44 vs. 4 ± 2.66 (*p*- 0.02). Serum lactic acid was higher in patients who developed AKI with a mean value of 6.8 ± 6.9 vs. 2.85 ± 1.55 mmol/l in the non-AKI group (*p*- 0.03). Lower hemoglobin levels and hyperlactic acidemia were significantly more prevalent in the AKI group. There were five deaths in this series (3.3%), and four of those (80%) were in the AKI group.

**Conclusion:** Using the KDIGO criteria, the incidence of AKI in infants and children following cardiac surgery was 9.3%. This is slightly lower than in previously published studies where the range was between 15 and 64%. Children with cyanotic cardiac disease, hyperlactic acidemia, and anemia were more prone to developing AKI. Identifying patients at risk might help decrease the risk of post-operative AKI.

## Introduction

The incidence of acute kidney injury (AKI) in pediatric patients following cardiac surgery varies between 15 and 64% (1–3), with a mortality rate of 10–89% (4) among those requiring dialysis (1, 3). The development of AKI is associated with higher mortality, more complicated hospital course, and higher risk of infection (5). This discrepancy in the incidence of AKI across studies is likely due to the inconsistent definitions used for AKI. The most commonly used classifications are those used by the Acute Kidney Injury Network (AKIN), Risk Injury Failure Loss End-stage renal disease (RIFLE), and pediatric modified Risk Injury Failure Loss End-stage renal disease (pRIFLE) (6, 7). The most recent and agreed-upon consensus definition is that of the Kidney Disease: Improving Global Outcomes (KDIGO) group.

One of the main challenges for managing AKI after cardiac surgery is creatinine elevation, which is not observed until 50% of kidney function is lost and often takes 24–48 h after the initial insult to rise, leading to delayed diagnosis (8). In several studies, other serum and urinary markers have been used for the early diagnosis of AKI. Among these, serum cystatin-C and urinary NGAL have become popular as functional biomarkers (9). Thus, it has been demonstrated that cystatin-C can be used as a functional biomarker of injury to diagnose AKI earlier than creatinine, with improved specificity and sensitivity.

Several mechanisms for AKI post-cardiac surgery have been described, including renal ischemia and reperfusion injury, maladaptive inflammatory response, oxidative stress, microemboli, and alteration in tubular cell metabolism (10, 11). Many risk factors for developing AKI have been identified, but only a few are modifiable. Several studies divide risk factors according to their temporal relation to the surgery. Preoperative risk factors include younger age at repair and higher surgical complexity (5); intraoperative factors include longer bypass time, hypotensive episodes, and duration of surgery (12). Post-operative factors include the use of nephrotoxic medications and systemic and wound infections (1).

Our study aimed to assess the incidence of AKI post-cardiac surgery in infants and children admitted to the pediatric intensive care unit (PICU) in a tertiary care center in a developing country, Lebanon, compared to the reported literature. We chose the KDIGO criteria for the definition of AKI. Early recognition of risk factors may allow us to develop strategies for identifying patients that might develop AKI, with a potential reduction in morbidity and mortality.

## Patients And Methods

We conducted a retrospective chart review of pediatric patients who underwent cardiac surgery between December 2015 and January 2017. All were admitted to the PICU at the American University of Beirut Medical Center (AUBMC) in Lebanon. The PICU at our institution admits patients aged from 28 days to 18 years. A team of dedicated pediatric and cardiac intensivists manage all the cardiac surgeries. The study was approved by the Institutional Review Board at the American University of Beirut (IRB# BIO-2018-0089). Eligible patients were infants, children, and adolescents with congenital heart disease who underwent surgical palliation or correction with or without cardiopulmonary bypass (CPB). Exclusion criteria were neonates with AKI, preexisting chronic kidney disease, and end-stage kidney disease undergoing dialysis; premature babies; surgery for patent ductus arteriosus; the use of ACE inhibitors preoperatively; and incomplete or missing medical records.

Data collected included age in years, gender, type of heart disease, prior cardiac surgeries (Table 1), exposure to nephrotoxic drugs, RACHS-1 category (Table 2), pre- and post-operative creatinine levels in mg/dl, exposure to contrast pre-/post-op, serum bicarbonate levels (reference range: 22–26 mmol/l), intraoperative systolic and diastolic blood pressure, bypass time in minutes, cross-clamp time in minutes, hemoglobin level (11.5–13.0 in mg/dl), ejection fraction post-cardiac surgery, plasma lactate levels (reference range: 0.5–2.5 mmol/l), and infections. Serum creatinine levels were measured twice daily post-cardiac surgery (reference range: 0.3–0.7 mg/dl for children <3 years and 0.5–1.0 mg/dl from 3 to 18 years). The baseline creatinine was the serum level obtained 48 h before the surgical intervention. AKI was considered if the serum creatinine level increased by ≥0.3 mg/dl from baseline within 48 h (13). The need for renal replacement therapy (continuous veno-venous hemodiafiltration, peritoneal, or hemodialysis), need for mechanical circulatory support, length of PICU and hospital stay, and in-hospital mortality were also recorded. All surgeries were performed by the same cardiac surgeon.

**Table 1:** Demographic data and patient characteristics (*N* = 150).

	**No AKI *N* = 136**	**AKI *N* = 14**	***P*-value**
**Gender**			
Male	77 (56.6)	9 (64.3)	0.78
Female	59 (43.4)	5 (35.7)	
Age (year)	4.49 ± 4.03	7.36 ± 5.79	0.03
Cyanotic	60 (44.1)	11 (78.6)	0.02
Previous cardiac surgery	90 (66.2)	12 (85.7)	0.23

**Table 2:** Type of surgeries and surgical characteristics of patients (*N* = 150).

	**No AKI *N* = 136**	**AKI *N* = 14**	***P*-value**
RACHS	2.51 ± 0.79	2.77 ± 0.58	0.11
Bypass time (min)	130.88 ± 85.64	132.67 ± 34.04	0.23
Cross clamp time (min)	79.04 ± 45.15	74.30 ± 28.60	0.91
**RACHS-1 score (*****n*****, %)**
Score 1	7(4.7%)
Score 2	75(50.0%)
Score 3	49(32.7%)
Score 4	19(12.7%)
**Type of surgery (*****n*****, %)**
Fontan procedure	21(14.0%)
Pulmonary valve repair	12(8.0%)
Mitral valve repair	9(6.0%)
ASD closure	7(4.7%)
AV repair	7(4.7%)
TOF repair	25(16.7%)
VSD closure	48(32.0%)

## Statistical Analysis

Descriptive statistics were carried using the mean and standard deviation for continuous variables, whereas numbers and percentages were categorical.

The association between AKI and categorical variables was carried out using the Fisher's exact test, whereas the Mann–Whitney *U*-test was used to associate with continuous ones. Data were analyzed using the Statistical Package for Social Sciences (SPSS, version 23), and *p* < 0.05 was considered to indicate statistical significance.

## Results

Our study included 200 children admitted to the PICU following cardiac surgery. Fifty patients were excluded because of incomplete data. Fourteen patients developed AKI as per the KDIGO criteria. Children who developed AKI were older, with a mean age of 7.4 years compared to 4.5 years in the non-AKI group (*p* = 0.03). Our study's age distribution was as follows: 6% were <1 year of age, 84% 1–10 years, and 10% 10–18 years. The male-to-female ratio was 1.34, and gender was not significantly different between the groups (*p* = 0.78), but this ratio was 1.8 in those developing AKI. Patients with cyanotic heart disease were more prone to develop AKI (78%) compared to non-cyanotic patients (44%) (*p*-0.02). Patients with elevated post-operative (Table 3) creatinine levels were at high risk of developing AKI. Thus, post-op serum creatinine was significantly higher in the AKI group, with an average of 2.25 mg/dl compared to 0.36 mg/dl in the non-AKI group (*p* < 0.0001). Pre-op serum creatinine levels were higher in the AKI group, with an average of 0.8 mg/dl compared to 0.38 mg/dl in the non-AKI group, but it was not statistically significant (*p* = 0.32). The RACHS-1 score was higher in the AKI group with a mean of 2.77 but did not reach statistical difference (*p* = 0.11). Serum lactic acid was significantly higher in patients who developed AKI with a mean value of 6.8 ± 6.9 vs. 2.85 ± 1.55 mmol/l in the non-AKI group (*p*- 0.03; Table 3). Preoperative hemoglobin levels were significantly lower in patients who developed (10.81 ± 1.93 gm/dl in AKI vs. 15.19 ± 1.46 gm/dl in non-AKI, *p* < 0.0001).

**Table 3:** Clinical and biochemical data of patients (*N* = 150).

	**No AKI *N* = 136**	**AKI *N* = 14**	***P*-value**
Hemoglobin	15.19 ± 1.46	10.81 ± 1.93	<0.0001
**EF**
<56	20 (14.7)	2 (14.3)	1.00
>56	116 (85.3)	12 (85.7)	
Use of contrast	8 (5.9)	2 (15.4)	0.21
Positive fluid balance	40	4	1.00
Use of nephrotoxic	46 (36.8)	7 (53.8)	0.25
Creatinine pre-op	0.38 ± 0.15	0.80 ± 1.38	0.32
Bicarbonate	30.45 ± 2.92	20.64 ± 3.15	0.89
SBP	104.40 ± 11.98	106.50 ± 12.35	0.42
DBP	60.69 ± 10.25	58.86 ± 8.41	0.70
Creatinine post-op	0.36 ± 0.14	2.25 ± 1.86	<0.0001
Lactic acid	2.85 ± 1.55	6.79 ± 6.91	0.03

None of the patients received blood transfusions before the surgical intervention. The ejection fraction was <56% in 20 patients in the non-AKI group and two patients in the AKI group (*p* = 1), and the ejection fraction was >56% in 116 in the non-AKI group and 12 in the AKI group (*p* = 1).

Patients with AKI were more acidotic at baseline, but lower bicarbonate levels were not associated with a higher risk of AKI (*p* = 0.89). The average systolic and diastolic blood pressure was not different between the groups. Bypass and cross-clamping time were similar in duration in both AKI and non-AKI groups with respective *p*-values of 0.23 and 0.91. Extracellular fluid overload did not pose a significant problem in our patients with AKI, since there was no difference in the occurrence from the non-AKI patients (Table 3). Oliguria (urine output <1 ml/kg/h) was present in only 3/14 patients with AKI.

The average mechanical ventilation period and total hospital stay in days were not different between the groups (*p* = 0.17 and 0.62, respectively). However, patients who developed AKI had a longer length of stay in the PICU (*p* = 0.02; Table 4).

**Table 4:** Clinical outcomes of patients (*N* = 150).

	**No AKI *N* = 136**	**AKI *N* = 14**	***P*-value**
Death	1 (0.7)	4 (28.6)	<0.0001
Ventilation days	1.60 ± 1.92	2.57 ± 2.93	0.17
Hospital days	9.84 ± 9.79	10.79 ± 7.85	0.62
Length of PICU days	2.56 ± 1.44	4.00 ± 2.66	0.02

Four of the 14 patients with AKI died (28.6%). There was only one death (0.7%) in the non-AKI group (*p* < 0.0001). One patient in the AKI group required peritoneal dialysis for 48 h, with subsequent improvement in kidney function. Two patients in the AKI group died from ischemic brain injury, and two from cardiogenic shock. The patient in the non-AKI group died from myocardial failure.

## Discussion

AKI is a common comorbidity following cardiac surgery and is associated with increased mortality in the adult and pediatric population. Our study aimed to assess the incidence of AKI post-cardiac surgery using the KDIGO criteria in infants and children admitted to the PICU in a tertiary care center.

The incidence of AKI in post-operative cardiac patients according to KDIGO criteria was 9.3%, which is less than in previously published similar studies where the range is between 15 and 64%. The incidence of pediatric AKI following heart surgery varies based on the different definitions used and the study population's age. In our study, we chose the KDIGO criteria, but other well-defined tools including the pRIFLE (pediatric Risk, Injury, Failure, Loss of function, and End-stage renal disease) criteria and the AKIN (acute kidney injury network) criteria have been used to diagnose AKI in pediatric patients.

Lex et al. (14) compared the three criteria used in pediatric patients and found that the AKIN criteria were the most specific, while those of pRIFLE were the most sensitive. They also reported that the KDIGO criteria fell somewhere in between. Furthermore, the latter was validated in the pediatric critical care population by Selewski et al. (15); thus, we opted for the KDIGO.

Congenital heart diseases (CHDs) have long been recognized as a potential cause of nephropathy. Patients with cyanotic heart disease are at a greater risk of developing AKI and even chronic kidney disease if correction is not performed in a timely manner. The presence of hypoxemia, secondary polycythemia, and abnormal arterial venous shunts might lead to changes in renal blood flow and intraglomerular hemodynamics, thus affecting renal function (16).

Several studies looking at the risk factors for developing AKI post-cardiac surgery have found that age is an important one and that infants <1 year of age are at higher risk (17, 18). In our series, only 6% of the patients were younger than 1 year. It seems that the reduced ability to adapt to post-CPB inflammation and ischemia–reperfusion injury of the kidney in younger patients makes them more prone to inflammatory and ischemic insults since the maximal glomerular filtration rate is not achieved until after 2 years of age (19, 20). Of note, Amini et al. (21) found no correlation between AKI and age younger than 1 year, but again, only 8% of patients in that series were below 1 year of age.

Like the study by Cardoso et al. (22), our study found that a higher post-operative lactate level—a sign of hypoperfusion—was an independent risk factor for AKI, confirming the theory of hypoperfusion as a proposed mechanism of AKI. Preoperatively, a higher serum creatinine level was detected in the patient population who went on to develop AKI, but this was not statistically significant (*p* = 0.32). The average RACHS-1 category did not reach statistical significance in our study. Although the RACHS-1 score has been associated with post-operative AKI (23), some studies failed to show this association (24). A possible interpretation could be the paucity of classified cases as high risk (RACHS-1–category−5, 6).

One of the well-described modifiable factors in the literature as an independent predictor of AKI is preoperative hemoglobin (25). Consistent with the literature (24), the patients who had preoperative anemia were more prone to developing AKI in our study. Patients with non-cyanotic heart disease but with low hemoglobin were more prone to develop AKI. Thus, correcting anemia in this group preoperatively might decrease the risk of developing AKI post-cardiac surgery. In agreement with published data, we found that AKI is associated with increased mortality and morbidity (26).

Several studies have shown that a longer CPB duration (>120 min) is associated with post-operative AKI (1, 24). CPB time may be associated with more severe ischemia and progressive inflammation, which increases the risk of AKI. Although in our patient population, CPB was >130 min, this did not impact which patients would go on to develop AKI. The same was applied for the cross-clamp time and ejection fraction at baseline; neither was found to be a predictor for AKI. We found no correlation between the use of nephrotoxic medications such as aminoglycosides, non-steroidal anti-inflammatory drugs, ACE inhibitors, and the risk for AKI as described in some studies (27). When needed, trough plasma levels of nephrotoxic drugs were measured frequently, limiting the risk of reaching high/toxic levels. An accurate fluid volume status evaluation is essential for appropriate therapy as inadequate assessment can result in either hyper- or hypovolemia. We evaluated the fluid balance status of our patients based on weight gain. A patient was considered fluid overloaded, in case of weight gain ≥5–7%, and or clinical signs of hypervolemia (edema or signs of pulmonary edema on chest X-ray). As for fluid overload, this did not contribute significantly to the prevalence and/or mortality in our group of patients with AKI. A possible explanation was the strict monitoring of the fluid balance and the use of loop diuretics when needed. We are aware of several confounders that might have influenced our results, among which the absence of enough data about the use of inotropes, intraoperative blood loss volume, chest tube loss, and correction of hemoglobin to the degree of cyanosis. However, being a retrospective study, it is difficult to measure all these variables routinely.

AKI has been associated with longer mechanical ventilation duration, intensive care unit (ICU) stay, and hospital stay. This coincides with our experience where the length of the PICU stay was higher in children in the AKI group. Nevertheless, despite a longer hospital stay, the difference between the AKI and the non-AKI groups regarding hospital stay was not statistically significant.

## Limitations

We acknowledge that the present study is not without limitations. First, this is a retrospective study conducted by paper chart review, so unmeasured or absence of data on potential confounding factors may have influenced the results. Also, since we excluded neonates and included only older children admitted to the PICU, this may have influenced our outcomes in getting a lower incidence of AKI.

## Conclusions

The incidence of AKI in infants and children following cardiac surgery in our series was 9.3%. This is somewhat lower than in previously published studies where the incidence ranged between 15 and 64%. Children with cyanotic cardiac disease, hyperlactic acidemia, and anemia were significantly more prone to developing AKI. Prospective studies are required to confirm these findings. Identifying patients at risk might help decrease the risk of post-operative AKI.

## Data Availability Statement

The raw data supporting the conclusions of this article will be made available by the authors, without undue reservation.

## Ethics Statement

The studies involving human participants were reviewed and approved by the Institutional Review Board at the American University of Beirut. Written informed consent from the participants legal guardian/next of kin was not required to participate in this study in accordance with the national legislation and institutional requirements. Written informed consent to participate in this study was provided by the participants' legal guardian/next of kin.

## Author Contributions

BA and GD collected the data and wrote the initial draft. KD helped in collecting the data. HT performed the statistical analysis. IE was who performed all operations in the department. MA, FB, and ZB were who followed patients before and after surgery. RS and JA followed children after cardiac surgery, during hospitalization in the intensive care unit. JD was who assisted in the urological care of the patients, data collection, and draft revision. MM was who followed children in the PICU and helped in literature review and article revision. SS helped in writing the article and followed children with AKI in the PICU. All authors contributed to the article and approved the submitted version.

## Conflict of Interest

The authors declare that the research was conducted in the absence of any commercial or financial relationships that could be construed as a potential conflict of interest.

## Publisher's Note

All claims expressed in this article are solely those of the authors and do not necessarily represent those of their affiliated organizations, or those of the publisher, the editors and the reviewers. Any product that may be evaluated in this article, or claim that may be made by its manufacturer, is not guaranteed or endorsed by the publisher.
